# MCSimMod: An R Package for Working with Ordinary Differential Equation Models Encoded in the MCSim Model Specification Language

**DOI:** 10.21105/joss.08492

**Published:** 2025-08-15

**Authors:** Dustin F. Kapraun, Todd J. Zurlinden, Ryan D. Friese, Andrew J. Shapiro

**Affiliations:** 1 U.S. Environmental Protection Agency, U.S.A.; 2 Pacific Northwest National Laboratory, U.S.A.

## Summary

Many physical and biological phenomena can be described using mathematical models based on ordinary differential equations (ODEs). In such a model, an ODE describes how a “state variable” changes (quantitatively) with respect to an independent variable (e.g., time or position). In general, an ODE model can include several state variables, each with its own ODE, so the model can be expressed as a system of ODEs. Thus, if *y* is a vector of *n* state variables, an ODE model that describes the state of the system at *t* (i.e., at a specific time or value of the independent variable) can be expressed as

(1) $$\frac{\text{d}}{\text{d}t}y(t)=f(y(t),\theta ,t),$$

where *f* is a vector-valued function (of dimension *n*) and *θ* is a vector of parameters (i.e., constants or variables other than the state variables and the independent variable).

In order to obtain a specific solution for a system of ODEs, one must know the initial state of the system,

(2) $$y_{0}=y(t_{0}),$$

where *t*_0_ is the inital value of the independent variable. Equation 2 is often described as a statement of the “initial conditions” of the system, and solving a system of ODEs subject to such initial conditions is called solving an initial value problem (IVP).

For the R programming language and environment (R Core Team, 2024), there are at least two packages available that facilitate solving of IVPs. The R package deSolve (Soetaert et al., 2010) can be used to solve IVPs for ODEs that have been encoded either in R or in a compiled language, such as C or Fortran. For models encoded in a compiled language, one can compile the model source code to generate machine code that typically executes much more quickly than R code, which must be “interpreted” anew each time it’s executed on a computer. The deSolve package includes functions that provide interfaces to well-documented, public-domain IVP solution routines implemented in FORTRAN, including four ODE integrators from the package ODEPACK, and in C, including solvers of the Runge-Kutta family. The R package mrgsolve (Baron, 2024) also includes functions that can be used to solve IVPs. These mrgsolve functions provide interfaces to IVP solution routines implemented in C++. Despite their different implementations, the packages deSolve and mrgsolve use many of the same underlying IVP solution algorithms.

We developed the R package MCSimMod to facilitate ODE modeling within the R environment. MCSimMod allows one to solve IVPs for ODE models that have been described in the MCSim (Bois, 2009) model specification language using ODE integration functions from the R package deSolve (Soetaert et al., 2010). This system enables users to take advantage of the flexibility and post hoc data analysis capabilities of the interpreted language R while also achieving computational speeds typically associated with compiled programming languages like C and FORTRAN. Furthermore, this system encourages modelers to use separate files for defining models and executing model simulations, which can, in turn, improve modularity and reusability of source code developed for modeling analyses. Examples of model specification files and vignettes that demonstrate how to use them to perform simulations are included in the MCSimMod package. While other packages, including deSolve (Soetaert et al., 2010), mrgsolve (Baron, 2024), and diffeqr (Rackauckas & Nie, 2017), facilitate ODE modeling in the R environment, MCSimMod is unique in that it allows users to work directly with ODE models encoded in the MCSim model specification language.

MCSimMod does not incorporate all features of the GNU MCSim software (Bois, 2009), which, in addition to allowing one to define and perform simulations with ODE models, includes functions for performing Monte Carlo (MC) simulations and generating Bayesian distributional parameter estimates via Markov chain Monte Carlo (MCMC) methods. Nevertheless, one can easily use MCSimMod to perform MC simulations by solving multiple ODE IVPs in which different (randomly selected) parameter values are used for each IVP and then analyzing the results. Also, one can use the R package FME (Soetaert & Petzoldt, 2010) in conjunction with MCSimMod to generate parameter estimates via multiple methods (including MCMC methods) and to perform sensitivity analyses.

## Statement of need

Physiologically based pharmacokinetic (PBPK) models, which are a class of ODE models that describe absorption, distribution, metabolism, and excretion of a substance by a biological organism, are frequently used to inform human health risk assessments for environmental chemicals and the development of pharmaceuticals. For many years, the programming language ACSL and the associated programming environment acslX were the tools of choice for many scientists and researchers that work with PBPK models, but in 2015, the company that maintained acslX announced that it would no longer support or sell the acslX software. Prior to the decline of acslX, some PBPK modelers used the free and open-source software tools R and MCSim (usually separately) to perform computational modeling work, but once acslX became unavailable, many PBPK modelers began using R and MCSim together to implement PBPK models. (See, for example, PBPK models published by Pearce et al. (2017); Bernstein et al. (2021); and Campbell et al. (2023).) R and MCSim each have benefits and drawbacks when it comes to working with ODE models. R is a flexible and popular programming language and environment for analyzing data and generating graphics. However, because R is in an interpreted language, R statements must be translated into machine instructions each time they are executed on a computer. Consequently, complex calculations (such as those associated with PBPK model simulations) encoded in R are generally performed relatively slowly. MCSim is a more specialized software tool designed for implementing and calibrating mathematical models – it is not a general purpose programming tool like R. However, MCSim takes advantage of compiled languages (as acslX did) to perform model simulations quickly, making it an appealing choice when one needs to perform many simulations, as is typically the case for MC analyses and MCMC parameter estimation. One can leverage the strengths of both R and MCSim by defining an ODE model in the relatively simple MCSim model specification language, translating the model to C using an MCSim utility (called “mod”), compiling the C model to obtain machine code, and then performing model simulations rapidly and easily by writing R scripts that make use of the compiled code through the deSolve package. Unfortunately, installing R, MCSim, and other required software and ensuring that they work together properly can be challenging and has presented obstacles for many in the PBPK modeling community. We developed the R package MCSimMod as an easy-to install, user-friendly software tool that takes advantage of the flexibility of R and the computational speed of MCSim to meet the needs of PBPK modelers (especially those already familiar with MCSim), but MCSimMod can be used to solve any IVP (for any type of ODE model) and it is therefore a valuable resource for anyone seeking to work with ODE models in R.
